# Treatment-related mortality in head and neck cancer patients receiving chemotherapy and radiation: results of a meta-analysis of published trials

**DOI:** 10.1177/17588359241288251

**Published:** 2025-01-10

**Authors:** Cristina Gurizzan, Michela Cinquini, Lorenzo Legramandi, Carlo Resteghini, Marco Siano, Cristiana Bergamini, Luigi Lorini, Davide Smussi, Alberto Paderno, Lisa Licitra, Paolo Bossi

**Affiliations:** Medical Oncology and Hematology Unit, Humanitas Cancer Center, IRCCS Humanitas Research Hospital, Rozzano, Milan, Italy; Laboratory of Methodology of Sistematic Reviews and Guidelines production, Department of Oncology, Istituto di Ricerche Farmacologiche Mario Negri IRCCS, Milano, Italy; Laboratory of Methodology of Sistematic Reviews and Guidelines production, Department of Oncology, Istituto di Ricerche Farmacologiche Mario Negri IRCCS, Milano, Italy; Medical Oncology and Hematology Unit, Humanitas Cancer Center, IRCCS Humanitas Research Hospital, Rozzano, Milan, Italy; Department of Biomedical Sciences – Humanitas University, Pieve Emanuele, Milan, Italy; Oncology Unit, Seeland Cancer Center, Biel, Switzerland; Head and Neck Medical Oncology Department, Fondazione IRCCS Istituto Nazionale dei Tumori, Milan, Italy; Medical Oncology and Hematology Unit, Humanitas Cancer Center, IRCCS Humanitas Research Hospital, Rozzano, Milan, Italy; Department of Medical and Surgical Specialties, Medical Oncology, Radiological Sciences and Public Health University of Brescia, ASST-Spedali Civili, Brescia, Italy; Department of Biomedical Sciences – Humanitas University, Pieve Emanuele, Milan, Italy; Otorhinolaryngology Head & Neck Surgery Unit, IRCCS Humanitas Research Hospital, Rozzano, Milan, Italy; Head and Neck Medical Oncology Department, Fondazione IRCCS Istituto Nazionale dei Tumori, Milan, Italy; Medical Oncology and Hematology Unit, Humanitas Cancer Center, IRCCS Humanitas Research Hospital, Via Alessandro Manzoni 56, 20089 Rozzano, Italy; Department of Biomedical Sciences – Humanitas University, Pieve Emanuele, Milan, Italy

**Keywords:** biostatistics, chemoradiotherapy (chemoradiation), clinical trials, combination therapy, meta-analysis, supportive care, systematic reviews

## Abstract

**Objectives:**

A combination of chemotherapy and radiotherapy is employed in the curative and postoperative treatment of locally advanced head and neck cancers (HNC). Integrated chemoradiation (CRT) treatments result in a non-negligible rate of severe toxic effects. Treatment-related death (TRD) is a crucial topic for physicians involved in the curative treatment of HNC. This meta-analysis aimed to better address TRD in locally advanced HNC patients treated with CRT through available and relevant literature.

**Methods:**

We performed a systematic review of the literature according to the PRISMA statement. The studies fulfilling these criteria included the following: concurrent or alternating CRT; both radical and postoperative settings; published from 2000 to 2020; involving 100+ patients; and available toxicity data. TRD was defined as death occurring from CRT start until a month from the end of CRT. Potential TRD predictors were evaluated.

**Results:**

In all, 65 studies were retrieved, with a total of 235 TRDs reported accounting for an overall risk rate of 1.4%. At meta-regression analysis, T stage and neutropenia grade >3 were potential predictors of higher TRD risk, both in univariate and multivariate analyses. Considering only the studies reporting at least one event, laryngeal/hypopharyngeal, oral cavity subsites and renal failure were significant predictors for TRD. The oropharyngeal subsite was protective in both analyses. None of the predictors proved to be independently correlated with TRD at multivariable analysis.

**Conclusion:**

CRT in HNC resulted in 1.4% of TRDs. TRD rate reduction may imply better patient selection and more intensive supportive care programs.

## Introduction

A combination of chemotherapy (CT) and radiotherapy (RT) is employed in the treatment of unresectable locally advanced head and neck cancers (HNC) or as an alternative to surgery for organ and function preservation.^1–3^ Moreover, chemoradiation (CRT) is the standard adjuvant postoperative treatment in resected tumors at high risk of recurrence.^4,5^

In all these conditions, integrated treatments have been shown to produce an advantage in survival as compared with radiotherapy alone.^6^

Beyond the possible combination strategies (sequential, concurrent, alternating), type of drugs, radiotherapy techniques, and facilities used, these integrated treatments result in greater patient exposure to severe toxic effects, in particular mucositis, radiation dermatitis, dysphagia, infections, and bone marrow suppression.^5,7^

HNC patients form a distinct cancer population with often prognostic unfavorable comorbidities prone to complications and higher toxicity, particularly under CRT. Treatment-related death (TRD) is a crucial topic for physicians involved in the curative treatment of HNC.^1,8,9^

Mortality of treated patients in the CRT arm of randomized-controlled trials is consistently described to be between 2% and 3%.^10^ However, in retrospective studies and case series in which the selection of patients and temporal TRD threshold tend to be less rigorous, the post-CRT early mortality rate seems higher reaching values varying from 5.4% up to 18.3%.^11–13^

Up to date, there are no consistent predictive factors able to identify a population at higher risk of death due to CRT; some reports showed that increasing age, stage, and performance status, as well as male sex, earlier treatment year, and cancer subsite are associated with higher TRD.^14,15^ Moreover, a meta-analysis of randomized trials showed a positive correlation between age and non-cancer-related mortality of integrated treatments or altered fractionation radiotherapy. Age above 70 years abolished the benefit from the administration of concurrent chemotherapy described in the general population.^16^ Also, the risk of dying of causes not related to cancer, including those related to the treatment, is about double in patients older than 70 years with respect to younger ones.^17^

We performed a meta-analysis of available and relevant literature on locally advanced HNC patients treated with CRT to better address the burden, causes, and potential predictive factors of TRD.

## Methods

This systematic review was conducted and reported following the PRISMA statement.^18^ Since there is no consensus on a temporal definition of TRD, we adopted the stricter criteria of Adelstein et al.^1^ defining TRD as death occurring from CRT start until a month from the end of CRT.

### Search strategy

We searched MEDLINE, EMBASE, and CENTAL using free-text words and Thesaurus terms specific to each database. We searched for publications containing the following items: “head and neck cancer,” “chemoradiation,” “chemo-radiotherapy,” “toxicity,” “toxic deaths,” and “treatment-related mortality” (Supplemental Material 1). Thereafter, we eliminated duplicates and filtered for studies fulfilling the following eligibility criteria: concurrent or alternating CRT; conducted in both radical and postoperative setting; published from January 1, 2000 to January 30, 2020; involving 100+ patients; with available toxicity data.

### Type of studies and eligibility

Phase II and III randomized controlled trials (RCTs), prospective and retrospective observational studies, and case series of HNC patients, matching the above-mentioned criteria, were considered for inclusion in the final analysis. The cutoff of 100+ patients was chosen since the literature on head and neck cancer treatment clearly states the importance of a number of patients treated to consider the quality of the treatment itself.

Case series were only considered if presenting homogeneous treatment modalities. Eligible studies should compare relevant strategies where at least one study arm performed CRT. In the case of studies with different treatment arms, each single eligible treatment arm (i.e. performing CRT) was considered separately in the statistical analysis. Matching studies should report toxicities according to the CTCAE (common toxicity criteria classification of Adverse Events) grading system, TRD, and described in the Methods, Results, or Discussion section of the abstract or the full publication. Only publications written in English were considered. Reviews, editorials, and conference abstracts were excluded.

Patients included in the examined studies were aged 18 years or older and had locally advanced HNSCC with stage III or IV disease, qualifying for curatively intended treatment with both chemo- and radiotherapy. Only studies including patients with squamous cell carcinoma histology involving at least one subsite of the following: oral cavity, oropharynx, hypopharynx, and larynx were considered. Studies including other subsites in addition to at least one of those just mentioned were also considered eligible.

Eligibility was independently assessed by five collaborators (P.B., C.G., C.R., C.B., and M.S.). Only publications with the longest follow-up were considered in case of more publications of the same study. Disagreements were resolved by consensus. Data extraction was performed by the same five collaborators and filled in a pre-specified data form (Supplemental Material 2).

The following study-specific parameters were recorded: name of collaborator, date of extraction, first author, year of publication, study design, country of origin or international setting, multicenter setting, study treatments, type and dose of treatments (CT and RT), schedule of CT, fractionation of RT, number of treatment arms, start and end of accrual, number of patients enrolled, minimal and maximal follow-up time, rate and type of G3/4 toxicity according to CTCAE, TRD rate, and cause of TRD.

Patient- and tumor-related parameters were also assessed and included in the pre-specified data form: mean and median age, gender, TNM stage, ethnicity, smoking status, alcohol consumption, performance status (ECOG), primary tumor site, and human papillomavirus (HPV) status.

### Quality assessment

Cochrane tool for risk of bias assessment of RCTs was used.^19^ We used the Newcastle–Ottawa Scale for observational studies.^20^ A table summarizing the quality assessment of included studies is produced as Supplemental Material 3.

### Statistical analysis

The primary outcome was the proportion of patients with the toxic death occurrence. The statistical analyses were performed in Stata Version 12 and SAS 9.3. Meta-analysis was performed using Multilevel mixed effects binomial regression, suggested in cases with a lot of proportions with zero events,^21^ and the standard errors and confidence intervals for a single proportion were derived. Heterogeneity was assessed using the *I*^2^ and the DerSimonian and Laird method with the Freeman-tukey double arcsine transformation.^21^ Meta-regression was used to explore potential sources of heterogeneity in the case of *I*^2^ > 25%.

The following parameters were considered as explanatory variables for TRD: study design, type of treatment, type of chemotherapy, induction treatment, year of publication, year of enrollment start, median age, gender, patients with curative treatment, tumor stage T3-4, nodal stage N2-3, oropharyngeal cancers, larynx or hypopharynx tumors, oral cavity and other subsites tumors, and the occurrence of grade >3 adverse events (i.e. neutropenia, anemia, weight loss, renal failure, infection/pneumonia, or mucositis).

Other important variables such as the percentage of smokers, alcohol abusers, and HPV-positive tumors were excluded since data were reported in less than 10 studies. Metastatic patients were excluded.

Univariable analysis was performed to test for significance. Variables with a *p*-value of ⩽0.05 in the univariable analyses were included in a multivariable model. A sensibility analysis was performed to validate the results. In the sensibility analysis were considered only the study without zero toxic death observed. The method used was the same as the principal analysis.

## Results

### Study selection

**Figure 1.**
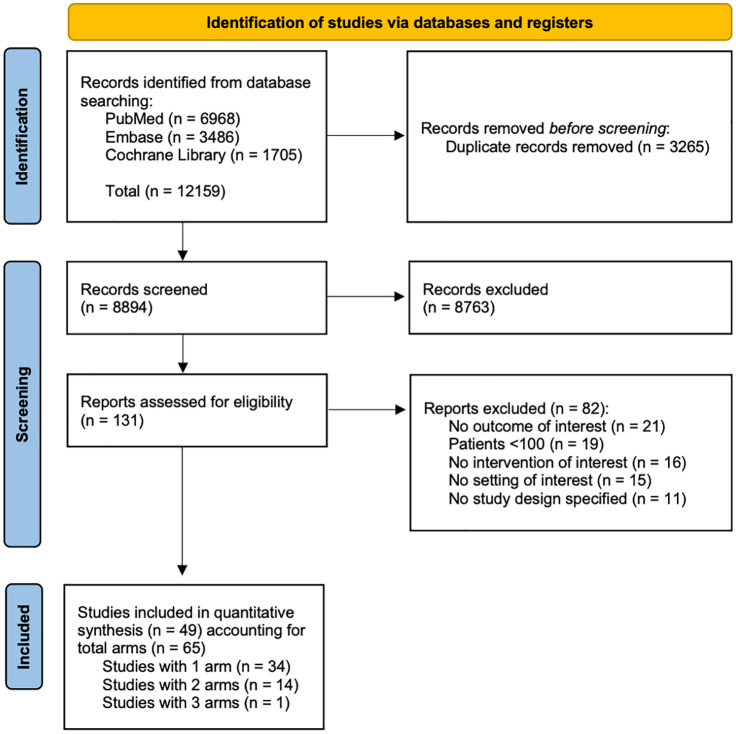
PRISMA flow diagram of paper selection.

As illustrated in Figure 1, of the 12,159 papers identified only 49 studies were included in quantitative synthesis, accounting for a total of 65 arms of treatment. Study characteristics, alongside patients’ characteristics, are shown in Table 1. Full references to the included studies are provided in Supplemental Material 4.

**Table 1.** Studies and patient characteristics of included studies in the final analysis.

Characteristics	Total *n* = 65 (%)
Type of study	
Observational	35 (53.8)
Experimental	30 (46.2)
Type of radiation treatment	
Standard fractionation	49 (75.4)
Altered fractionation	16 (24.6)
Induction chemotherapy treatment	
No	56 (86.2)
Yes	9 (13.8)
Year of publication	
Median (Q1–Q3)	2011 (2005–2014)
Min–Max	2000–2019
Missing	0
Start of enrollment (year)	
Median (Q1–Q3)	1999 (1995–2005)
Min–Max	1989–2013
Missing	2
Median age	
Median	57.5 (50.0–72.0)
Missing	19
Percentage of patients with tumor stage T3-4	
Mean (SD)	74.2 (15.1)
Missing	8
Percentage of patients with nodal stage N2-3	
Mean (SD)	57.3 (19.4)
Missing	9
Percentage of patients with oropharynx tumor	
Mean (SD)	49.6 (28.1)
Percentage of patients with larynx or hypopharynx tumor	
Mean (SD)	33.3 (25.0)
Percentage of patients with oral cavity tumor	
Mean (SD)	13.1 (17.2)
Percentage of patients with other primary tumor subsite	
Mean (SD)	4.0 (13.4)

### Patient characteristics and toxicities

A total of 10,480 patients were considered; the median age was 58.4 years (range, 50–72 years) with a male-to-female rate of 4.6:1. Most patients underwent exclusive curative treatment with only 10.9% undergoing postoperative therapy. A summary of patient and disease characteristics is found in Table 1. As depicted in Table 2, the most frequent severe toxicities (grade >3) reported were mucositis and neutropenia.

**Table 2.** Most commonly reported severe toxicities in the studies included for final analysis.

Toxicity	Total *n* = 65 studies (total patients 10,480)
Percentage of patients with grade ⩾3 neutropenia	
Mean (SD)	26.4 (24.6)
Missing	18
Percentage of patients with grade ⩾3 anemia	
Mean (SD)	6.8 (6.2)
Missing	39
Percentage of patients with grade ⩾3 weight loss	
Mean (SD)	21.5 (22.6)
Missing	50
Percentage of patients with grade ⩾3 renal failure	
Mean (SD)	11.0 (15.9)
Missing	46
Percentage of patients with grade ⩾3 infection/pneumonia	
Mean (SD)	15.8 (16.0)
Missing	46
Percentage of patients with grade ⩾3 mucositis	
Mean (SD)	46.6 (24.8)
Missing	16

### Estimated risk for severe toxicity and mortality

**Figure 2.**
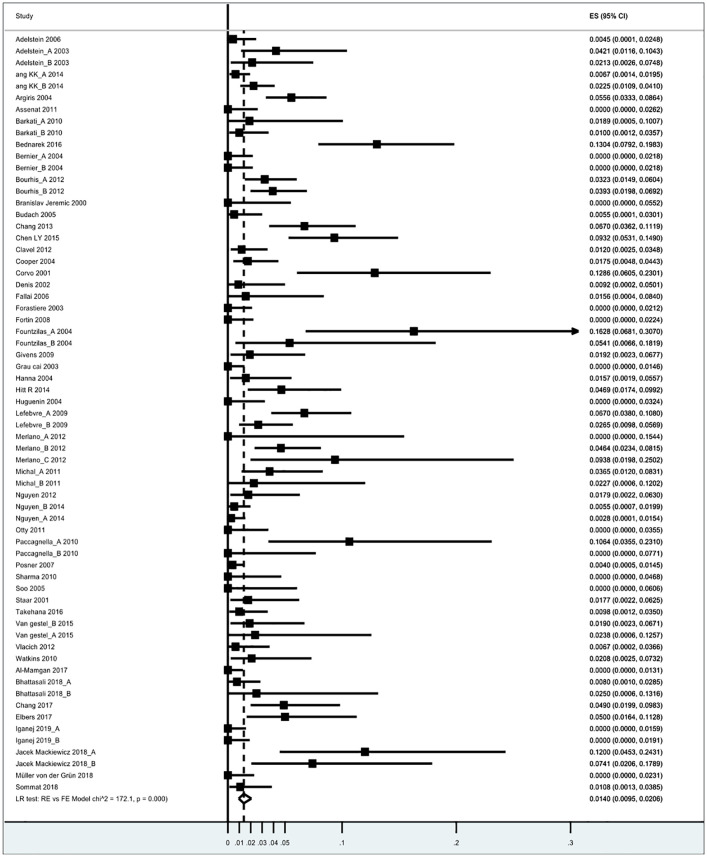
Forest plot of estimated treatment-mortality risk rate.

A total of 235 TRDs were reported with an overall risk rate of 1.4% (95% CI 0.95%–2.06%) with an *I*^2^ = 79.6 (Figure 2).

A meta-regression analysis was performed, so to evaluate whether baseline factors and/or dynamic factors occurring during treatment (type and grade of toxicities), study design and year of publication, and treatment characteristics could affect toxic death incidence. With such analysis, no difference was revealed between TRD reported in observational studies compared to experimental studies 0.81 (95% CI 0.39–1.71) (*p* = 0.585); similarly, year of publication 1.02 (95% CI 0.95–1.10) (*p* = 0.510) and type of treatment 0.98 (95% CI 0.39–2.49) (*p* = 0.960) did not affect TRD. Significant factors at baseline predicting TRD were primary tumor stage and oropharyngeal primary tumor site, while among reported toxicities only neutropenia grade >3 predicted TRD (Table 3). Table 3 also shows the multivariable analysis conducted on all the studies with a minimum of variables considered and including only statistically significant variables in the univariable analysis. The increment of the percentage of patients with grade >3 neutropenia and tumor stage shows a statistically significant increment in the risk rate of TRD.

**Table 3.** Univariable and multivariable meta-regression using mixed effects binomial-normal model.

Univariable meta-regression	*N*	*p*-value of variable	OR (95% CI)
Study design	65	0.585	
Observational			Reference
Experimental			0.81 (0.39–1.71)
Type of treatment	63	0.960	
Standard fractionation			Reference
Altered fractionation			0.98 (0.39–2.49)
Type of chemotherapy	63	0.146	
Mono-chemotherapy			Reference
Polychemotherapy			1.75 (0.82–3.70)
Induction chemotherapy treatment	63	0.910	
No			Reference
Yes			1.07 (0.32–3.64)
Year of publication	65	0.510	1.02 (0.95–1.10)
Year of beginning enrollment	63	0.691	1.01 (0.95–1.08)
Median age	46	0.691	0.98 (0.87–1.09)
Increment by 5% regarding the percentage of patients:			
Males	65	0.374	1.09 (0.90–1.32)
With curative treatment	61	0.432	1.03 (0.95–1.12)
With tumor stage T3-4	57	**0.046**	1.15 (1.00–1.33)
With tumor stage N2-3	56	0.240	1.08 (0.95–1.21)
With oropharynx tumor	65	**0.048**	0.94 (0.88–0.99)
With a larynx or hypopharynx tumor	65	0.150	1.05 (0.98–1.13)
With a tumor in the oral cavity	65	0.264	1.06 (0.96–1.18)
With other tumors	65	0.844	0.99 (0.86–1.13)
With grade ⩾3 neutropenia	47	**0.035**	1.10 (1.01–1.19)
With grade ⩾3 anemia	26	0.314	1.25 (0.81–1.94)
With grade ⩾3 weight loss	15	0.152	0.88 (0.74–1.05)
With grade ⩾3 renal failure	19	0.394	1.11 (0.87–1.42)
With grade ⩾3 infection/pneumonia	19	0.580	1.07 (0.85–1.34)
With grade ⩾3 mucositis	49	0.590	1.02 (0.95–1.10)
Multivariable meta-regression	*p*-value of variable		OR (95% CI)
Increment by 5% regarding the percentage of patients:			
With grade ⩾3 neutropenia	**0.018**		1.12 (1.02–1.23)
With oropharynx tumor	0.707		0.98 (0.88–1.09)
With tumor stage T3-4	**0.009**		1.24 (1.06–1.46)

*N*, number of analyzed studies; OR, odds ratio. In bold *p*-values <0.05.

Considering only studies with at least one event with a toxic fatality, laryngeal/hypopharyngeal and oral cavity tumor subsite and higher tumor stage proved to be significant predictors for TRD, while oropharyngeal subsite represented a protective subgroup. Moreover, renal failure showed to be another predictor, with an OR of 1.22 (95% CI 1.00–1.44) (*p* = 0.042) as illustrated in Table 4. However, none of the predictors found for studies with at least one toxic fatal event proved to be independently correlated with TRD at multivariable analysis, with only a trend toward significance for oral cavity subsite (renal failure was excluded for low numbers of studies; Table 4). To be noted, multivariate analysis was performed only for those variables being reported in all the 48 studies reporting at least one event with toxic fatality.

**Table 4.** Univariable and multivariable meta-regression using mixed effects binomial-normal model on studies with at least one patient with toxic death.

Univariable meta-regression	*N*	*p*-value of variable	OR (95% CI)
Type of study	48	0.620	
Observational			Reference
Experimental			0.86 (0.46–1.59)
Type of treatment	46	0.449	
Standard fractionation			Reference
Altered fractionation			0.75 (0.36–1.57)
Type of chemotherapy	46	0.978	
Mono-chemotherapy			Reference
Polychemotherapy			0.99 (0.52–1.86)
Induction chemotherapy treatment	46	0.414	
No			Reference
Yes			0.67 (0.25–1.76)
Year of publication	48	0.836	1.01 (0.95–1.07)
Year of beginning enrollment	47	0.817	1.01 (0.96–1.06)
Median age	32	0.335	0.95 (0.87–1.05)
Increment by 5% regarding the percentage of patients:			
Males	48	0.056	1.16 (0.99–1.36)
With curative treatment	45	0.564	0.98 (0.91–1.05)
With tumor stage T3-4	40	**0.045**	1.13 (1.00–1.27)
With tumor stage N2-3	40	0.164	0.93 (0.83–1.03)
With oropharynx tumor	48	**0.002**	0.92 (0.88–0.97)
With a larynx or hypopharynx tumor	48	**0.051**	1.06 (1.00–1.12)
With a tumor in the oral cavity	48	**0.026**	1.10 (1.01–1.19)
With other tumors	48	0.629	0.87 (0.88–1.09)
With grade ⩾3 neutropenia	34	0.108	1.05 (0.99–1.13)
With grade ⩾3 anemia	20	0.539	1.12 (0.77–1.61)
With grade ⩾3 weight loss	11	0.301	0.93 (0.80–1.07)
With grade ⩾3 renal failure	13	**0.042**	1.22 (1.00–1.47)
With grade ⩾3 infection/pneumonia	14	0.989	1.01 (0.83–1.21)
With grade ⩾3 mucositis	39	0.936	1.00 (0.94–1.07)
Multivariable meta-regression	***p***-value of variable		OR (95% CI)
Increment by 5% regarding the percentage of patients:			
With oropharynx tumor	0.778		0.99 (0.89–1.09)
With a larynx or hypopharynx tumor	0.234		1.07 (0.96–1.18)
With a tumor in the oral cavity	0.086		1.11 (0.99–1.25)

*N*, number of analyzed studies; OR, odds ratio.

## Discussion

In our analysis, we were able to retrieve 65 studies fulfilling all the eligibility criteria, aimed at selecting studies of patients treated with CRT, both with exclusive and postoperative intent, with at least 100 patients included. These characteristics allow us to evaluate the rate of early mortality and to define possible correlations with baseline or therapeutic factors.

We decided to analyze the trials with a stringent criterion of “early mortality,” that is, all the deaths occurring during treatment and in the following 30 days.^1^ We chose this criterion to ideally restrict the events to those related to treatment-related toxicities and try to avoid the inclusion of early deaths due to cancer progression. In this regard, the literature is quite heterogeneous; trials reported a rate of early death varying from 5% to 15% when considering a longer timeframe after treatment ends (90 days).^22–24^

Therefore, applying our strict criteria, we were able to find an overall risk rate of 1.4% of early deaths in the current meta-analysis. The high heterogeneity index should also be considered, explained by the quite long time considered and the wide variety of subsites and types of patients included.

Our finding was definitely in accordance with the pooled TRD rate (1.44%) reported for locally advanced non-small-cell lung cancer patients treated with concurrent CRT in randomized clinical trials.^25^ In this paper, Zhao et al. demonstrated no differences in terms of TRD risk for concurrent CRT regimens compared to non-concurrent ones; also, no difference was found in TRDs between high and low radiation doses during CRT using a threshold of 66 Gy.

In the present analysis, male patients were predominant (male to female ratio 4.6:1), reflecting the fourfold higher incidence of head and neck cancer in men.^26^ However, women have been reported to bear a worse prognosis compared to men suffering from HNC with a higher cumulative incidence of cancer-specific death.^27^ Also, women are less likely to receive chemotherapy combined with radiotherapy in a definitive setting and are underrepresented in clinical trials.^28^ This disparity could potentially explain the predominance of male patients in our analysis.

Moreover, we aimed to explore potential predictors of TRD, both considered at baseline and dynamically during treatment. Given the underreporting of the cause of death in most clinical trials, we based our analysis on severe toxicities. It is conceivable that treatment-related toxicities probably represent at the same time a proxy and a cause of TRD. To support this fact, a large work conducted on the National Cancer Institute’s Surveillance, Epidemiology, and End Results (SEER) registry demonstrated that patients at higher risk of non-cancer death largely belong to two subgroups: those with chronic comorbidities (mainly cardiovascular and cerebrovascular disease) and those with acute, iatrogenic, and treatment-induced infections.^29^

Analyzing potential predictors of TRD, we found differences according to tumor subsite. Oropharyngeal cancer (OPC) patients present a lower TRD rate, while patients suffering from larynx/hypopharynx and oral cavity cancers seem to be more prone to TRD.

This discrepancy reflects a different natural history of diseases arising from different subsites. Oropharyngeal subsite represents a proxy for HPV-related cancer, whose prevalence has been steadily increasing since 2000.^30^ HPV-related OPC represents a peculiar subpopulation compared to HPV-unrelated HNC patients, with fewer comorbidities, less exposure to smoking and alcohol, better nutritional status, higher socioeconomic status,^31^ and better prognosis in terms of both overall survival and disease-free survival.^30^ A meta-analysis showed that patients with HPV-related OPC have a 28% reduced risk of death compared with non-HPV-related OPC.^32^ On the other hand, larynx and hypopharynx subsites represent two subsites more prone to aspiration, with a higher rate of baseline aspiration often asymptomatic.^33^ Aspiration can result in pneumonia, with significant morbidity and mortality. Nguyen et al. retrospectively evaluated swallowing function in 63 HNC patients who underwent concurrent chemoradiation therapy and reported a 9% fatal aspiration pneumonia.^34^ Similarly, oral cavity cancer patients are frailer than the others, with more comorbidities and more alcohol/tobacco consumption, so the difference seen here in tumor type as a risk factor for TRD could related to underlying medical conditions.

Another predictor found to be linked to an increased risk of TRD was advanced T stage (i.e. T3-4) High disease burden has been associated with severe late toxicity, due to more extensive target volumes at radiation therapy,^35^ thus implying higher severity of mucositis, painful swallowing, dysphagia or aphagia, weight loss, overall clinical condition impairment, and increased rates of potentially life-threatening infections. Even if theoretically the risk of carotid blowout due to radiotherapy is a risk for head and neck cancer patients, this is almost always confined to patients receiving re-irradiation with high cumulative doses to vascular structures. Instead, the risk of a carotid blowout in the first treatment course is mainly due to the direct invasion of the tumor into the wall of the vessel.

We also found that the development of grade 3 or higher neutropenia predicted TRD. This may be attributed to the increased risk of potentially fatal infections and sepsis in the presence of a reduced leukocyte count. Finally, the development of grade 3 or higher platinum-related renal toxicity has been associated with TRD. It is conceivable that patients at greatest risk of platinum renal failure are those with more comorbidities and frailties at baseline. Of note, comorbidities not only increase the risk of oncologic treatment toxicity by themselves but may also interact with chemotherapeutic drugs, thereby further increasing toxicity. Takeuchi and colleagues showed an increased reporting odds ratio for patients concomitantly treated with cisplatin and a combination of antihypertensive drugs (calcium channel blockers and renin–angiotensin system inhibitors).^36^

Among factors not proved to be predictive of TRD, of note N stage and weight loss during treatment. Both were found to be associated with severe late RT-related toxicity (i.e. grade ⩾3 toxicity starting 3 months after the end of RT and/or potential treatment-related death within 3 years of randomization) in a retrospective analysis from the previously reported data of the randomized phase III trial SAKK 10/94.^37^ This discrepancy can be partially attributed to the fact that advanced nodal status may be a proxy for HPV, while weight loss suffers from a paucity of data reporting. In 65 trials included in the present analysis, the median percentage of patients with severe weight loss was 13% (range 2%–67%), with 50% of missing data. The data are largely underestimated since severe weight loss during radiotherapy is reported to occur in almost one-half of HNC patients (48.7%). Also, the risk of weight loss is higher in patients with larynx and oral cavity cancer subsite.^38^

We only evaluated data from clinical trials; however, in the unselected real-world patient population, results may be different. A recently published review has witnessed the gap that divides patients enrolled in randomized clinical trials from those every day treated in clinical practice.^39^ HNC patients selected for clinical trials present a better performance status and are younger than the median age reported by cancer registries.

In our analysis, no differences in TRD rate were found according to the year of publication were found. However, on one side, we should have expected an increase in TRD due to a steady increase in the use of induction chemotherapy from 2000 till the publication of negative randomized trials.^40,41^ On the other hand, thanks to the increasing number of HPV+ oropharyngeal cancer diagnoses,^30^ together with an improvement in the learning curve of treatment adverse events and advances in radiotherapy techniques,^42^ a downward trend in TRD in relation to more recent publication years. It should be considered, nevertheless, that the concept of treatment de-escalation started only in the late 2010s, so not being captured by our analysis. Hence, it is possible to hypothesize that these opposing factors balanced each other resulting in a non-effect on the relationship between TRD rate and year of publication.

In 2019, Zapata et al. reported the incidence of non-cancer mortalities in a homogeneous cohort of locally advanced HNC patients treated with radiotherapy and systemic treatment. Out of 284 patients, 153 patients died and of those 14 patients (9%) died as a consequence of acute treatment toxicity.^43^ Reasons for this discrepancy could be attributed to less stringent criteria for administering CRT in real life; less selected population; less codified supportive care treatment; older patients; and different sociocultural levels and insurance status. Gaubatz et al.^44^ demonstrated that racial and socioeconomic disparities among HNC patients have a non-negligible impact on 90-day mortality. In particular, blacks, males, uninsured, and living in lower median income zip code patients presented a greater 90-day mortality rate. In addition, in a retrospective analysis of pharyngeal and laryngeal squamous cell carcinoma patients treated with definitive radiotherapy, performance status >1, hemoglobin <100 g/l, weight <60 kg, age >80 years, and presence of multiple comorbidities were demonstrated to be associated with a higher risk of early death.^23^ Also, a scoring system capable of predicting 90-day mortality after completion of CRT in HNC patients was proposed in a prognostic study conducted on the Taiwan Cancer Registry Database.^45^ This predictive score was based on clinical factors including the patient’s age (⩾50 or ⩾70) and comorbidities (pneumonia, sepsis, hemiplegia, moderate or severe renal disease, leukemia, and presence of other metastatic solid cancers).

The main limit of the present study is that not all studies included in our analysis report all relevant clinical data, such as performance status, nutritional status, weight loss, and socioeconomic status, all recognized as related to an increased risk of treatment-related toxicity. Also, the data about HPV status in OPC are lacking, thus not allowing any granular evaluation. Moreover, causes of death are never fully reported in clinical studies and therefore it is very complicated to trace a true relationship of causality between treatment and patients’ death. We acknowledge the difficulty of ascertaining the TRD using only a temporal criterion, as we almost always faced the issue of the absence of a clear explanation of the causal event. Finally, as abovementioned, we only evaluated data from clinical trials that do not perfectly match with the real-world population.

We, therefore, advocate for a consistent reporting of treatment-related deaths and their causes to better manage and select HNC patients candidates to receive chemoradiation, both with exclusive and postoperative intent. In addition, the implementation of adequate supportive care since the very beginning of the treatment course is crucial to avoid and control any possible treatment-related toxicity.

## Supplemental Material

sj-docx-1-tam-10.1177_17588359241288251 – Supplemental material for Treatment-related mortality in head and neck cancer patients receiving chemotherapy and radiation: results of a meta-analysis of published trials Supplemental material, sj-docx-1-tam-10.1177_17588359241288251 for Treatment-related mortality in head and neck cancer patients receiving chemotherapy and radiation: results of a meta-analysis of published trials by Cristina Gurizzan, Michela Cinquini, Lorenzo Legramandi, Carlo Resteghini, Marco Siano, Cristiana Bergamini, Luigi Lorini, Davide Smussi, Alberto Paderno, Lisa Licitra and Paolo Bossi in Therapeutic Advances in Medical Oncology

sj-docx-2-tam-10.1177_17588359241288251 – Supplemental material for Treatment-related mortality in head and neck cancer patients receiving chemotherapy and radiation: results of a meta-analysis of published trials Supplemental material, sj-docx-2-tam-10.1177_17588359241288251 for Treatment-related mortality in head and neck cancer patients receiving chemotherapy and radiation: results of a meta-analysis of published trials by Cristina Gurizzan, Michela Cinquini, Lorenzo Legramandi, Carlo Resteghini, Marco Siano, Cristiana Bergamini, Luigi Lorini, Davide Smussi, Alberto Paderno, Lisa Licitra and Paolo Bossi in Therapeutic Advances in Medical Oncology

sj-docx-3-tam-10.1177_17588359241288251 – Supplemental material for Treatment-related mortality in head and neck cancer patients receiving chemotherapy and radiation: results of a meta-analysis of published trials Supplemental material, sj-docx-3-tam-10.1177_17588359241288251 for Treatment-related mortality in head and neck cancer patients receiving chemotherapy and radiation: results of a meta-analysis of published trials by Cristina Gurizzan, Michela Cinquini, Lorenzo Legramandi, Carlo Resteghini, Marco Siano, Cristiana Bergamini, Luigi Lorini, Davide Smussi, Alberto Paderno, Lisa Licitra and Paolo Bossi in Therapeutic Advances in Medical Oncology

sj-docx-4-tam-10.1177_17588359241288251 – Supplemental material for Treatment-related mortality in head and neck cancer patients receiving chemotherapy and radiation: results of a meta-analysis of published trials Supplemental material, sj-docx-4-tam-10.1177_17588359241288251 for Treatment-related mortality in head and neck cancer patients receiving chemotherapy and radiation: results of a meta-analysis of published trials by Cristina Gurizzan, Michela Cinquini, Lorenzo Legramandi, Carlo Resteghini, Marco Siano, Cristiana Bergamini, Luigi Lorini, Davide Smussi, Alberto Paderno, Lisa Licitra and Paolo Bossi in Therapeutic Advances in Medical Oncology

## Declarations

**Ethics approval and consent to participate:** Not applicable.

**Consent for publication:** Not applicable.

**Availability of data and materials:** The data underlying this article are available in the article and its online Supplemental Material.
