# Supplementary material for: Treatment-related mortality in head and neck cancer patients receiving chemotherapy and radiation: results of a meta-analysis of published trials
Source: Ther Adv Med Oncol. 2025 Jan 10;17:17588359241288251. doi: 10.1177/17588359241288251 (PMC11724409; doi:10.1177/17588359241288251)
Supplement: sj-docx-1-tam-10.1177_17588359241288251 – Supplemental material for Treatment-related mortality in head and neck cancer patients receiving chemotherapy and radiation: results of a meta-analysis of published trials [file sj-docx-1-tam-10.1177_17588359241288251.docx]

**MEDLINE (Pubmed) (01/01/2000 to 30/01/2020)**

(((((((("Head and Neck Neoplasms"[Mesh]) OR (("oral cavity"[Title/Abstract]) AND (Tumor*[Title/Abstract] OR cancer*[Title/Abstract] OR neoplasm*[Title/Abstract]))) OR ((hypopharyngeal[Title/Abstract]) AND ((Tumor*[Title/Abstract] OR cancer*[Title/Abstract] OR neoplasm*[Title/Abstract])))) OR ((otorhinolaryngologic[Title/Abstract]) AND ((Tumor*[Title/Abstract] OR cancer*[Title/Abstract] OR neoplasm*[Title/Abstract])))) OR ((nose [Title/Abstract] OR "nose pharyngeal"[Title/Abstract] OR pharyngeal[Title/Abstract]) AND ((Tumor*[Title/Abstract] OR cancer*[Title/Abstract] OR neoplasm*[Title/Abstract])))) OR ((laryngeal[Title/Abstract]) AND ((Tumor*[Title/Abstract] OR cancer*[Title/Abstract] OR neoplasm*[Title/Abstract])))) OR ((oropharyngeal[Title/Abstract] OR rhinopharyngeal[Title/Abstract]) AND ((Tumor*[Title/Abstract] OR cancer*[Title/Abstract] OR neoplasm*[Title/Abstract])))) OR ((head[Title/Abstract] OR neck[Title/Abstract]) AND ((Tumor*[Title/Abstract] OR cancer*[Title/Abstract] OR neoplasm*[Title/Abstract])))) AND ((("Chemoradiotherapy"[Mesh]) OR "Chemoradiotherapy, Adjuvant"[Mesh]) OR (Chemoradiotherap*[Title/Abstract] OR Radiochemotherap*[Title/Abstract] OR "Adjuvant Chemoradiotherapies"[Title/Abstract] OR "Adjuvant Chemoradiotherapy"[Title/Abstract] OR "Adjuvant Radiochemotherapy"[Title/Abstract] OR "Adjuvant Radiochemotherapies"[Title/Abstract])) Filters: Adult: 19+ years, from 2000/1/1 - 2020/1/30

**Records: 6968**

**Embase (01/01/2000 to 30/01/2020)**

#1 'head and neck tumor'/exp/mj

#2 ('oral cavity' OR hypopharyngeal OR otorhinolarygologic OR laryngeal OR nose) AND pharyngeal OR pharyngeal OR oropharyngeal OR rhinopharyngeal OR head OR neck:ti,ab

#3 tumor* OR cancer* OR neoplasm*:ti,ab #4 #2 AND #3

#5 #1 OR #4

#6 'chemoradiotherapy'/exp/mj OR 'adjuvant chemoradiotherapy'/exp/mj

#7 chemoradiotherap* OR radiochemotherap* OR 'adjuvant chemoradiotherapies' OR 'adjuvant chemoradiotherapy' OR 'adjuvant radiochemotherapy' OR 'adjuvant radiochemotherapies':ti,ab #8 #6 OR #7

#9 #5 AND #8

#10 #5 AND #8 AND ([adult]/lim OR [aged]/lim OR [very elderly]/lim)

#11 #5 AND #8 AND ([adult]/lim OR [aged]/lim OR [very elderly]/lim) AND [2000-2020]/py #12 #11 AND [embase]/lim NOT ([embase]/lim AND [medline]/lim)

**Records: 3486**

**Cochrane Central Register of Controlled Trials (CENTRAL; 2020, Issue 1) in the Cochrane Library (searched 30 january 2020)**

#1 MeSH descriptor: [Head and Neck Neoplasms] explode all trees

#2 ("oral cavity" OR hypopharyngeal OR otorhinolarygologic OR laryngeal OR nose OR "nose pharyngeal" OR pharyngeal OR oropharyngeal OR rhinopharyngeal OR head OR neck):ti,ab,kw

#3 (Tumor* OR cancer* OR neoplasm*):ti,ab,kw #4 #2 AND #3

#5 #1 OR #4

#6 MeSH descriptor: [Chemoradiotherapy] explode all trees

#7 MeSH descriptor: [Chemoradiotherapy, Adjuvant] explode all trees

#8 (Chemoradiotherap* OR Radiochemotherap* OR “Adjuvant Chemoradiotherapies” OR “Adjuvant Chemoradiotherapy” OR “Adjuvant Radiochemotherapy” OR “Adjuvant Radiochemotherapies”):ti,ab,kw #9 #6 OR #7 OR #8

#10 #5 AND #9 with Publication Year from 2000 to 2020

**Records: 1705**
