# Supplementary material for: Treatment-related mortality in head and neck cancer patients receiving chemotherapy and radiation: results of a meta-analysis of published trials
Source: Ther Adv Med Oncol. 2025 Jan 10;17:17588359241288251. doi: 10.1177/17588359241288251 (PMC11724409; doi:10.1177/17588359241288251)
Supplement: sj-docx-2-tam-10.1177_17588359241288251 – Supplemental material for Treatment-related mortality in head and neck cancer patients receiving chemotherapy and radiation: results of a meta-analysis of published trials [file sj-docx-2-tam-10.1177_17588359241288251.docx]

| **C Coding** | | | | | | | | | |
| --- | --- | --- | --- | --- | --- | --- | --- | --- | --- |
| C1 | Study Number |  | | | | | | | |
| C2 | Evaluator |  | | | | | | | |
| C3 | Date of Extraction |  | | | | | | | |
| **DD Descriptive Data** | | | | | | | | | |
| DD1 | First Author |  | | | | | | | |
| DD2 | Year of Publication |  | | | | | | | |
| DD3 | Title |  | | | | | | | |
| DD4 | Study Design, if RCT  Description of trial design (i.e parallel, cross-over, cluster, placebo-controlled, ect.) |  | | | | | | | |
| DD5 | Study design | Cohort study | | | | | Case-control study | | |
| DD6 | Study design | Case series | | | | |  | | |
| DD7 |  |  | | |  | | |  | |
| DD8 | Country: | | | International | | | | | Not stated |
| DD9 | Setting: | | | | | | | | |
| **Inclusion** | | | Yes | | | NO | | | |
| If NO, reason of exclusion: | | | | | | | | | |
| **GN General Notes** | | | | | | | | | |
|  | | | | | | | | | |

| DD10 | Multi-center* | Yes | If yes # | No | Not stated |
| --- | --- | --- | --- | --- | --- |
|  |  |  | | | |
|  |  |  | | | |
|  |  |  | | | |
|  |  |  | | | |
|  |  |  | | | |
|  |  |  |  |  |  |
|  |  |  |  |  |  |
|  | | | | | |

|  | | | | | | | | |
| --- | --- | --- | --- | --- | --- | --- | --- | --- |
| **TX Treatment** | | | | | | | | |
| **Treatment A (TXA):** | | | | | | | | |
| **Treatment B (TXB):** | | | | | | | | |
| **Treatment C (TXC):** | | | | | | | | |
| **Treatment D (TXD):** | | | | | | | | |
|  | | | | | | | | |
|  | |  | Dose | | Route of admistration | | Frequency | |
| TXA | |  |  | |  | |  | |
| TXB | |  |  | |  | |  | |
| TXC | |  |  | |  | |  | |
| TXD | |  |  | |  | |  | |
| **Other treatments:** | | | | | | | | |
|  | | | | | | | | |
| **POP Population** | | | | | | | | |
| POP1 | Initial time of patient accrual | | |  | | | | |
| POP2 | Final time of patient accrual | | |  | | | | |
| POP3 | Number of patients enrolled (total) | | |  | | | | |
| POP4 | Number of treatment arms | | |  | | | | |
| POP5 | Crossover design | | | Yes | | | NO | |
|  |  | | | Treatment A | | Treatment B | Treatment C | Treatment D |
| POP6 | Number of patients enrolled | | |  | |  |  |  |
| POP7 | Mean age and (SD) | | |  | |  |  |  |
| POP8 | Median age and (range) | | |  | |  |  |  |
| POP9 | Male | | |  | |  |  |  |
| POP10 | Female | | |  | |  |  |  |
| POP11 | Min and Max follow-up | | |  | |  |  |  |
|  | T stage | | |  | |  |  |  |
| POP12 | 1 | | |  | |  |  |  |
| POP12 | 2 | | |  | |  |  |  |
| POP12 | 3 | | |  | |  |  |  |

| POP12 | 4 |  |  |  |  |
| --- | --- | --- | --- | --- | --- |
|  | N stage |  |  |  |  |
| POP13 | 0 |  |  |  |  |
| POP14 | 1 |  |  |  |  |
| POP15 | 2A |  |  |  |  |
| POP16 | 2B |  |  |  |  |
| POP17 | 2C |  |  |  |  |
| POP18 | 3 |  |  |  |  |
|  | M stage |  |  |  |  |
| POP19 | 0 |  |  |  |  |
| POP20 | 1 |  |  |  |  |
|  | Ethnic origin |  |  |  |  |
| POP21 | Caucasian |  |  |  |  |
| POP22 | Asian |  |  |  |  |
| POP23 | Other |  |  |  |  |
|  | Smoking status |  |  |  |  |
| POP24 | Never smoker |  |  |  |  |
| POP25 | Pipe/cigarette smoking |  |  |  |  |
| POP26 | <10 pack-year |  |  |  |  |
| POP27 | >10 pack-year |  |  |  |  |
|  | Alcohol intake |  |  |  |  |
| POP28 | None |  |  |  |  |
| POP29 | Occasional |  |  |  |  |
| POP30 | Moderate |  |  |  |  |
| POP31 | Heavy |  |  |  |  |
|  | Primary site |  |  |  |  |
| POP32 | Oropharynnx |  |  |  |  |
| POP33 | Larynx |  |  |  |  |
| POP34 | Hypopharynx |  |  |  |  |
| POP35 | Nasopharynx |  |  |  |  |
| POP36 | Oral Cavity |  |  |  |  |
| POP37 | Other |  |  |  |  |
|  | Performance status |  |  |  |  |
| POP38 | 0 |  |  |  |  |
| POP39 | 1 |  |  |  |  |
| POP40 | 2 |  |  |  |  |
|  | HPV status |  |  |  |  |
| POP41 | POSITIVE |  |  |  |  |
| POP42 | NEGATI VE |  |  |  |  |
|  |  |  |  |  |  |

|  | Treatment characteristic | |  | |  |  |  |
| --- | --- | --- | --- | --- | --- | --- | --- |
| POP43 | Type of concomitant chemotherapy | |  | |  |  |  |
| POP44 | Post operative treatment | |  | |  |  |  |
| POP45 | Exclusive treatment | |  | |  |  |  |
| POP46 | Induction chemotherapy | |  | |  |  |  |
| **OC Outcomes** | | | | | | | |
|  | | | Treatment A | Treatment B | | Treatment C | Treatment D |
|  | | **Toxic death** |  |  | |  |  |
| OC1 | | Patients analysed |  |  | |  |  |
| OC2 | | Number of patients with event |  |  | |  |  |
|  | | **Sub-acute death (30-90 days from**  **end of treatment)** |  |  | |  |  |
| OC3 | | Patients analysed |  |  | |  |  |
| OC4 | | Number of patients with event |  |  | |  |  |
|  | |  |  |  | |  |  |
| **Notes Cause of death :** | | | | | | | |
| **AE Adverse events** | | | | | | | |
|  | |  |  | | | | |
| AE1 | | % Grade 3/4 Neutropenia |  |  | |  |  |
| AE2 | | % Grade 3/4 Anemia |  |  | |  |  |
| AE3 | | % Grade 3/4 Weight loss |  |  | |  |  |
| AE4 | | % Grade 3/4 renal failure |  |  | |  |  |
| AE5 | | % Grade 3/4 infection/pneumonia |  |  | |  |  |
| AE6 | | % Grade 3/4 mucositis |  |  | |  |  |
| AE7 | | % Grade 3/4 thrombosis / pulmonary  embolism |  |  | |  |  |
| AE8 | | % Grade 3/4 bleeding |  |  | |  |  |
| AE9 | | % Any Grade 3/4 |  |  | |  |  |
|  | |  |  |  | |  |  |
|  | | | | | | | |
|  | | | | | | | |

| **QA Quality Assessment** | | | | |
| --- | --- | --- | --- | --- |
| **Only for RCTs** | | | Description | Judgment * |
| QA1 | Selection bias | Was the allocation sequence adequately generated? |  |  |
| QA2 | Selection bias | Was the allocation sequence adequately concealed? |  |  |
|  | Performance bias | Did the researchers rule out any impact from concurrent intervention or an intended  exposure that might bias results? |  |  |
|  | Attrition bias | Was attrition (overall or  differential nonresponse, dropout, loss to follow-up, or exclusion of participants) a concern? |  |  |
|  | Attrition bias | If attrition, were missing data handled appropriately (e.g., intention-to-treat analysis and imputation)? |  |  |
|  | Detection bias | Was the length of follow-up  different between the groups? |  |  |
|  | Reporting bias | Were potential outcomes prespecified by the  researchers? Are all prespecified outcomes reported? |  |  |
| **Only for CCTs or Cohort (or Cross-sectional)** | | |  |  |
|  | Selection bias | Were participants analysed  within the groups they were originally assigned to? |  |  |
|  | Selection bias | Did the strategy for  recruiting participants into the study differ across study groups? |  |  |
|  | Performance bias | Did the researchers rule out  any impact from concurrent intervention or an intended exposure that might bias  results? |  |  |
|  | Attrition bias | Was attrition (overall or  differential nonresponse, dropout, loss to follow-up, or exclusion of participants) a concern? |  |  |

|  | Attrition bias | If attrition, were missing data handled appropriately (e.g., intention-to-treat analysis and imputation)? |  |  |
| --- | --- | --- | --- | --- |
|  | Detection bias | Was the length of follow-up different between the groups? |  |  |
|  | Detection bias | Were confounding variables assessed using valid and reliable measures,  implemented consistently across all study participants? |  |  |
|  | Reporting bias | Were potential outcomes  prespecified by the researchers? Are all prespecified outcomes  reported? |  |  |
| **Only for Case-control** | | |  |  |
|  | Selection bias | Were cases and controls selected appropriately? (e.g. appropriate diagnostic criteria or definitions, equal application of exclusion criteria to cases and controls, sampling not influenced by exposure  status) |  |  |
|  | Performance bias | Did the researchers rule out any impact from concurrent intervention or an intended exposure that might bias  results? |  |  |
|  | Attrition bias | Was attrition (overall or differential nonresponse, dropout, loss to follow-up, or exclusion of participants)  a concern? |  |  |
|  | Attrition bias | If attrition, were missing data handled appropriately  (e.g., intention-to-treat analysis and imputation)? |  |  |
|  | Detection bias | Was the time period  between the intervention/exposure and outcome the same for cases  and controls? |  |  |
|  | Detection bias | Were confounding variables  assessed using valid and reliable measures, implemented consistently across all study participants? |  |  |
|  | Reporting bias | Were potential outcomes prespecified by the researchers? Are all prespecified outcomes  reported? |  |  |

***Judgmen**t: Adequate. Inadequate or Unclear
