# Supplementary material for: Treatment-related mortality in head and neck cancer patients receiving chemotherapy and radiation: results of a meta-analysis of published trials
Source: Ther Adv Med Oncol. 2025 Jan 10;17:17588359241288251. doi: 10.1177/17588359241288251 (PMC11724409; doi:10.1177/17588359241288251)
Supplement: sj-docx-3-tam-10.1177_17588359241288251 – Supplemental material for Treatment-related mortality in head and neck cancer patients receiving chemotherapy and radiation: results of a meta-analysis of published trials [file sj-docx-3-tam-10.1177_17588359241288251.docx]

| **Adequate** | **Inadequate** | **Unclear** |
| --- | --- | --- |
| **Randomized Controlled Trial – n tot 19** | | |
| 13/19 | -- | 6/19  4 selection and detection bias  (Adelstein 2003; Jeremic 2000; Budach 2005; Staar 2001)  1 performance and attrition bias  (Corvò 2001)  1 selection bias  (Fountzilas 2004) |
| **Case-control – n tot 4** | | |
| 4/4 | -- | -- |
| **Case series or Cohort – n tot 26** | | |
| 22/26 | 3/26  1 detection, selection and attrition bias  (Bednarek 2016)  1 selection and detection bias  (Chen 2015)  1detection, attrition and performance bias  (Müller von der Grün 2018) | 1/26  1 selection, detection and reporting bias (Sommat 2018) |
