# Supplementary material for: Treatment-related mortality in head and neck cancer patients receiving chemotherapy and radiation: results of a meta-analysis of published trials
Source: Ther Adv Med Oncol. 2025 Jan 10;17:17588359241288251. doi: 10.1177/17588359241288251 (PMC11724409; doi:10.1177/17588359241288251)
Supplement: sj-docx-4-tam-10.1177_17588359241288251 – Supplemental material for Treatment-related mortality in head and neck cancer patients receiving chemotherapy and radiation: results of a meta-analysis of published trials [file sj-docx-4-tam-10.1177_17588359241288251.docx]

**References of included studies**

Adelstein DJ, Saxton JP, Rybicki LA, Esclamado RM, Wood BG, Strome M, Lavertu P, Lorenz RR, Carroll MA. Multiagent concurrent chemoradiotherapy for locoregionally advanced squamous cell head and neck cancer: mature results from a single institution. J Clin Oncol. 2006 Mar 1;24(7):1064-71. doi: 10.1200/JCO.2005.01.5867. PMID: 16505425.

Adelstein DJ, Li Y, Adams GL, Wagner H Jr, Kish JA, Ensley JF, Schuller DE, Forastiere AA. An intergroup phase III comparison of standard radiation therapy and two schedules of concurrent chemoradiotherapy in patients with unresectable squamous cell head and neck cancer. J Clin Oncol. 2003 Jan 1;21(1):92-8. doi: 10.1200/JCO.2003.01.008. PMID: 12506176.

Ang KK, Zhang Q, Rosenthal DI, Nguyen-Tan PF, Sherman EJ, Weber RS, Galvin JM, Bonner JA, Harris J, El-Naggar AK, Gillison ML, Jordan RC, Konski AA, Thorstad WL, Trotti A, Beitler JJ, Garden AS, Spanos WJ, Yom SS, Axelrod RS. Randomized phase III trial of concurrent accelerated radiation plus cisplatin with or without cetuximab for stage III to IV head and neck carcinoma: RTOG 0522. J Clin Oncol. 2014 Sep 20;32(27):2940-50. doi: 10.1200/JCO.2013.53.5633. PMID: 25154822; PMCID: PMC4162493.

Argiris A, Brockstein BE, Haraf DJ, Stenson KM, Mittal BB, Kies MS, Rosen FR, Jovanovic B, Vokes EE. Competing causes of death and second primary tumors in patients with locoregionally advanced head and neck cancer treated with chemoradiotherapy. Clin Cancer Res. 2004 Mar 15;10(6):1956-62. doi: 10.1158/1078-0432.ccr-03-1077. PMID: 15041712.

Assenat E, Thezenas S, Flori N, Pere-Charlier N, Garrel R, Serre A, Azria D, Senesse P. Prophylactic percutaneous endoscopic gastrostomy in patients with advanced head and neck tumors treated by combined chemoradiotherapy. J Pain Symptom Manage. 2011 Oct;42(4):548-56. doi: 10.1016/j.jpainsymman.2011.01.009. Epub 2011 Apr 7. PMID: 21477980.

Barkati M, Fortin B, Soulières D, Clavel S, Després P, Charpentier D, Tabet JC, Guertin L, Olivier MJ, Coulombe G, Donath D, Nguyen-Tan PF. Concurrent chemoradiation with carboplatin-5-fluorouracil versus cisplatin in locally advanced oropharyngeal cancers: is more always better? Int J Radiat Oncol Biol Phys. 2010 Feb 1;76(2):410-6. doi: 10.1016/j.ijrobp.2009.02.034. Epub 2009 May 8. PMID: 19427745.

Bednarek C, Nguyen TV, Puyraveau M, Bonnet É, Lescut N, Azélie C, Miny J, Mauvais O, Maurina T, Tochet F, Bosset JF, Thariat J, Sun XS. Implementation of intensity-modulated radiotherapy for head and neck cancers in routine practice. Cancer Radiother. 2017 Feb;21(1):21-27. doi: 10.1016/j.canrad.2016.07.103. Epub 2016 Dec 27. PMID: 28034680.

Bernier J, Domenge C, Ozsahin M, Matuszewska K, Lefèbvre J-L, Greiner RH, et al. Postoperative Irradiation with or without Concomitant Chemotherapy for Locally Advanced Head and Neck Cancer. N Engl J Med 2004;350:1945–52. https://doi.org/10.1056/NEJMoa032641.

Bourhis J, Sire C, Graff P, Grégoire V, Maingon P, Calais G, Gery B, Martin L, Alfonsi M, Desprez P, Pignon T, Bardet E, Rives M, Geoffrois L, Daly-Schveitzer N, Sen S, Tuchais C, Dupuis O, Guerif S, Lapeyre M, Favrel V, Hamoir M, Lusinchi A, Temam S, Pinna A, Tao YG, Blanchard P, Aupérin A. Concomitant chemoradiotherapy versus acceleration of radiotherapy with or without concomitant chemotherapy in locally advanced head and neck carcinoma (GORTEC 99-02): an open-label phase 3 randomised trial. Lancet Oncol. 2012 Feb;13(2):145-53. doi: 10.1016/S1470-2045(11)70346-1. Epub 2012 Jan 18. PMID: 22261362.

Jeremic B, Shibamoto Y, Milicic B, Nikolic N, Dagovic A, Aleksandrovic J, Vaskovic Z, Tadic L. Hyperfractionated radiation therapy with or without concurrent low-dose daily cisplatin in locally advanced squamous cell carcinoma of the head and neck: a prospective randomized trial. J Clin Oncol. 2000 Apr;18(7):1458-64. doi: 10.1200/JCO.2000.18.7.1458. PMID: 10735893.

Budach V, Stuschke M, Budach W, Baumann M, Geismar D, Grabenbauer G, Lammert I, Jahnke K, Stueben G, Herrmann T, Bamberg M, Wust P, Hinkelbein W, Wernecke KD. Hyperfractionated accelerated chemoradiation with concurrent fluorouracil-mitomycin is more effective than dose-escalated hyperfractionated accelerated radiation therapy alone in locally advanced head and neck cancer: final results of the radiotherapy cooperative clinical trials group of the German Cancer Society 95-06 Prospective Randomized Trial. J Clin Oncol. 2005 Feb 20;23(6):1125-35. doi: 10.1200/JCO.2005.07.010. PMID: 15718308.

Chang P-H, Yeh K-Y, Huang J-S, Lai C-H, Wu T-H, Lan Y-J, et al. Pretreatment performance status and nutrition are associated with early mortality of locally advanced head and neck cancer patients undergoing concurrent chemoradiation. Eur Arch Oto-Rhino-Laryngology 2013;270:1909–15. https://doi.org/10.1007/s00405-012-2290-2.

Chen LY, Huang CC, Tsou YA, Bau DT, Tsai MH. Prognostic factor of severe complications in patients with hypopharyngeal cancer with primary concurrent chemoradiotherapy. Anticancer Res. 2015 Mar;35(3):1735-41. PMID: 25750336.

Clavel S, Charron MP, Bélair M, Delouya G, Fortin B, Després P, Soulières D, Filion E, Guertin L, Nguyen-Tan PF. The role of computed tomography in the management of the neck after chemoradiotherapy in patients with head-and-neck cancer. Int J Radiat Oncol Biol Phys. 2012 Feb 1;82(2):567-73. doi: 10.1016/j.ijrobp.2010.11.066. Epub 2011 Feb 9. PMID: 21310545.

Cooper JS, Pajak TF, Forastiere AA, Jacobs J, Campbell BH, Saxman SB, Kish JA, Kim HE, Cmelak AJ, Rotman M, Machtay M, Ensley JF, Chao KS, Schultz CJ, Lee N, Fu KK; Radiation Therapy Oncology Group 9501/Intergroup. Postoperative concurrent radiotherapy and chemotherapy for high-risk squamous-cell carcinoma of the head and neck. N Engl J Med. 2004 May 6;350(19):1937-44. doi: 10.1056/NEJMoa032646. PMID: 15128893.

Corvò R, Benasso M, Sanguineti G, Lionetto R, Bacigalupo A, Margarino G, Pallestrini E, Merlano M, Vitale V, Rosso R. Alternating chemoradiotherapy versus partly accelerated radiotherapy in locally advanced squamous cell carcinoma of the head and neck: results from a phase III randomized trial. Cancer. 2001 Dec 1;92(11):2856-67. doi: 10.1002/1097-0142(20011201)92:11<2856::aid-cncr10132>3.0.co;2-6. PMID: 11753959.

Denis F, Garaud P, Bardet E, Alfonsi M, Sire C, Germain T, Bergerot P, Rhein B, Tortochaux J, Calais G. Final results of the 94-01 French Head and Neck Oncology and Radiotherapy Group randomized trial comparing radiotherapy alone with concomitant radiochemotherapy in advanced-stage oropharynx carcinoma. J Clin Oncol. 2004 Jan 1;22(1):69-76. doi: 10.1200/JCO.2004.08.021. Epub 2003 Dec 2. PMID: 14657228.

Fallai C, Bolner A, Signor M, Gava A, Franchin G, Ponticelli P, Taino R, Rossi F, Ardizzoia A, Oggionni M, Crispino S, Olmi P. Long-term results of conventional radiotherapy versus accelerated hyperfractionated radiotherapy versus concomitant radiotherapy and chemotherapy in locoregionally advanced carcinoma of the oropharynx. Tumori. 2006 Jan-Feb;92(1):41-54. doi: 10.1177/030089160609200108. PMID: 16683383.

Forastiere AA, Goepfert H, Maor M, Pajak TF, Weber R, Morrison W, et al. Concurrent Chemotherapy and Radiotherapy for Organ Preservation in Advanced Laryngeal Cancer. N Engl J Med 2003;349:2091–8. https://doi.org/10.1056/NEJMoa031317.

Fortin A, Caouette R, Wang CS, Vigneault E. A comparison of treatment outcomes by radiochemotherapy and postoperative radiotherapy in locally advanced squamous cell carcinomas of head and neck. Am J Clin Oncol. 2008 Aug;31(4):379-83. doi: 10.1097/COC.0b013e318165c036. PMID: 18845998.

Fountzilas G, Ciuleanu E, Dafni U, Plataniotis G, Kalogera-Fountzila A, Samantas E, Athanassiou E, Tzitzikas J, Ciuleanu T, Nikolaou A, Pantelakos P, Zaraboukas T, Zamboglou N, Daniilidis J, Ghilezan N. Concomitant radiochemotherapy vs radiotherapy alone in patients with head and neck cancer: a Hellenic Cooperative Oncology Group Phase III Study. Med Oncol. 2004;21(2):95-107. doi: 10.1385/MO:21:2:095. PMID: 15299181.

Givens DJ, Karnell LH, Gupta AK, Clamon GH, Pagedar NA, Chang KE, Van Daele DJ, Funk GF. Adverse events associated with concurrent chemoradiation therapy in patients with head and neck cancer. Arch Otolaryngol Head Neck Surg. 2009 Dec;135(12):1209-17. doi: 10.1001/archoto.2009.174. PMID: 20026818.

Grau C, Johansen LV, Hansen HS, Andersen E, Godballe C, Andersen LJ, Hald J, Møller H, Overgaard M, Bastholt L, Greisen O, Harbo G, Hansen O, Overgaard J. Salvage laryngectomy and pharyngocutaneous fistulae after primary radiotherapy for head and neck cancer: a national survey from DAHANCA. Head Neck. 2003 Sep;25(9):711-6. doi: 10.1002/hed.10237. PMID: 12953306.

Hanna E, Alexiou M, Morgan J, Badley J, Maddox AM, Penagaricano J, Fan CY, Breau R, Suen J. Intensive chemoradiotherapy as a primary treatment for organ preservation in patients with advanced cancer of the head and neck: efficacy, toxic effects, and limitations. Arch Otolaryngol Head Neck Surg. 2004 Jul;130(7):861-7. doi: 10.1001/archotol.130.7.861. PMID: 15262764.

Hitt R, Grau JJ, López-Pousa A, Berrocal A, García-Girón C, Irigoyen A, Sastre J, Martínez-Trufero J, Brandariz Castelo JA, Verger E, Cruz-Hernández JJ; Spanish Head and Neck Cancer Cooperative Group (TTCC). A randomized phase III trial comparing induction chemotherapy followed by chemoradiotherapy versus chemoradiotherapy alone as treatment of unresectable head and neck cancer. Ann Oncol. 2014 Jan;25(1):216-25. doi: 10.1093/annonc/mdt461. Epub 2013 Nov 19. PMID: 24256848.

Huguenin P, Beer KT, Allal A, Rufibach K, Friedli C, Davis JB, Pestalozzi B, Schmid S, Thöni A, Ozsahin M, Bernier J, Töpfer M, Kann R, Meier UR, Thum P, Bieri S, Notter M, Lombriser N, Glanzmann C. Concomitant cisplatin significantly improves locoregional control in advanced head and neck cancers treated with hyperfractionated radiotherapy. J Clin Oncol. 2004 Dec 1;22(23):4665-73. doi: 10.1200/JCO.2004.12.193. Epub 2004 Nov 8. Erratum in: J Clin Oncol. 2005 Jan 1;23(1):248. PMID: 15534360.

Lefebvre JL, Rolland F, Tesselaar M, Bardet E, Leemans CR, Geoffrois L, Hupperets P, Barzan L, de Raucourt D, Chevalier D, Licitra L, Lunghi F, Stupp R, Lacombe D, Bogaerts J, Horiot JC, Bernier J, Vermorken JB; EORTC Head and Neck Cancer Cooperative Group; EORTC Radiation Oncology Group. Phase 3 randomized trial on larynx preservation comparing sequential vs alternating chemotherapy and radiotherapy. J Natl Cancer Inst. 2009 Feb 4;101(3):142-52. doi: 10.1093/jnci/djn460. Epub 2009 Jan 27. PMID: 19176454; PMCID: PMC2724854.

Merlano MC, Monteverde M, Colantonio I, Denaro N, Lo Nigro C, Natoli G, Giurlanda F, Numico G, Russi E. Impact of age on acute toxicity induced by bio- or chemo-radiotherapy in patients with head and neck cancer. Oral Oncol. 2012 Oct;48(10):1051-1057. doi: 10.1016/j.oraloncology.2012.05.001. Epub 2012 Jun 2. PMID: 22658677.

Michal SA, Adelstein DJ, Rybicki LA, Rodriguez CP, Saxton JP, Wood BG, Scharpf J, Ives DI. Multi-agent concurrent chemoradiotherapy for locally advanced head and neck squamous cell cancer in the elderly. Head Neck. 2012 Aug;34(8):1147-52. doi: 10.1002/hed.21891. Epub 2011 Oct 22. PMID: 22021098.

Nguyen NP, Vock J, Chi A, Vinh-Hung V, Dutta S, Ewell L, Jang S, Betz M, Almeida F, Miller M, Davis R, Sroka T, Vo RP, Karlsson U, Vos P. Impact of intensity-modulated and image-guided radiotherapy on elderly patients undergoing chemoradiation for locally advanced head and neck cancer. Strahlenther Onkol. 2012 Aug;188(8):677-83. doi: 10.1007/s00066-012-0125-0. Epub 2012 Jun 3. PMID: 22659942.

Nguyen-Tan PF, Zhang Q, Ang KK, Weber RS, Rosenthal DI, Soulieres D, Kim H, Silverman C, Raben A, Galloway TJ, Fortin A, Gore E, Westra WH, Chung CH, Jordan RC, Gillison ML, List M, Le QT. Randomized phase III trial to test accelerated versus standard fractionation in combination with concurrent cisplatin for head and neck carcinomas in the Radiation Therapy Oncology Group 0129 trial: long-term report of efficacy and toxicity. J Clin Oncol. 2014 Dec 1;32(34):3858-66. doi: 10.1200/JCO.2014.55.3925. Epub 2014 Nov 3. PMID: 25366680; PMCID: PMC4239304.

Otty Z, Skinner MB, Dass J, Collins M, Mooi J, Thuraisingam K, Sabesan S. Efficacy and tolerability of weekly low-dose cisplatin concurrent with radiotherapy in head and neck cancer patients. Asia Pac J Clin Oncol. 2011 Sep;7(3):287-92. doi: 10.1111/j.1743-7563.2011.01405.x. PMID: 21884441.

Paccagnella A, Mastromauro C, D'Amanzo P, Ghi MG. Induction chemotherapy before chemoradiotherapy in locally advanced head and neck cancer: the future? Oncologist. 2010;15 Suppl 3:8-12. doi: 10.1634/theoncologist.2010-S3-08. PMID: 21036883.

Posner MR, Hershock DM, Blajman CR, Mickiewicz E, Winquist E, Gorbounova V, Tjulandin S, Shin DM, Cullen K, Ervin TJ, Murphy BA, Raez LE, Cohen RB, Spaulding M, Tishler RB, Roth B, Viroglio Rdel C, Venkatesan V, Romanov I, Agarwala S, Harter KW, Dugan M, Cmelak A, Markoe AM, Read PW, Steinbrenner L, Colevas AD, Norris CM Jr, Haddad RI; TAX 324 Study Group. Cisplatin and fluorouracil alone or with docetaxel in head and neck cancer. N Engl J Med. 2007 Oct 25;357(17):1705-15. doi: 10.1056/NEJMoa070956. PMID: 17960013.

Sharma A, Jagadesan P, Chaudhari P, Das S, Bhaskar S, Thakar A, Sharma A, Mohanti BK. Six-year analysis of compliance to weekly concurrent chemoradiotherapy in head and neck carcinomas. Clin Otolaryngol. 2016 Oct;41(5):442-7. doi: 10.1111/coa.12580. Epub 2016 Feb 11. PMID: 26523400.

Soo KC, Tan EH, Wee J, Lim D, Tai BC, Khoo ML, Goh C, Leong SS, Tan T, Fong KW, Lu P, See A, Machin D. Surgery and adjuvant radiotherapy vs concurrent chemoradiotherapy in stage III/IV nonmetastatic squamous cell head and neck cancer: a randomised comparison. Br J Cancer. 2005 Aug 8;93(3):279-86. doi: 10.1038/sj.bjc.6602696. PMID: 16012523; PMCID: PMC2361563.

Staar S, Rudat V, Stuetzer H, Dietz A, Volling P, Schroeder M, Flentje M, Eckel HE, Mueller RP. Intensified hyperfractionated accelerated radiotherapy limits the additional benefit of simultaneous chemotherapy--results of a multicentric randomized German trial in advanced head-and-neck cancer. Int J Radiat Oncol Biol Phys. 2001 Aug 1;50(5):1161-71. doi: 10.1016/s0360-3016(01)01544-9. Erratum in: Int J Radiat Oncol Biol Phys 2001 Oct 1;51(2):569. PMID: 11483325.

Takehana K, Kodaira T, Tachibana H, Kimura K, Shimizu A, Makita C, Tomita N, Nishikawa D, Suzuki H, Hirakawa H, Hanai N, Hasegawa Y. Retrospective analysis of the clinical efficacy of definitive chemoradiotherapy for patients with hypopharyngeal cancer. Jpn J Clin Oncol. 2016 Apr;46(4):344-9. doi: 10.1093/jjco/hyv216. Epub 2016 Jan 29. PMID: 26826721; PMCID: PMC4886140.

Van Gestel D, Van den Weyngaert D, De Kerf G, De Ost B, Vanderveken O, Van Laer C, Specenier P, Geussens Y, Wouters K, Meulemans E, Cheung KJ, Grégoire V, Vermorken JB. Helical tomotherapy in head and neck cancer: a European single-center experience. Oncologist. 2015 Mar;20(3):279-90. doi: 10.1634/theoncologist.2014-0337. Epub 2015 Feb 11. PMID: 25673104; PMCID: PMC4350799.

Vlacich G, Diaz R, Thorpe SW, Murphy BA, Kirby W, Sinard RJ, Shakhtour B, Shyr Y, Murphy P, Netterville JL, Yarbrough WG, Cmelak AJ. Intensity-modulated radiation therapy with concurrent carboplatin and paclitaxel for locally advanced head and neck cancer: toxicities and efficacy. Oncologist. 2012;17(5):673-81. doi: 10.1634/theoncologist.2011-0396. Epub 2012 May 1. PMID: 22550060; PMCID: PMC3360907.

Watkins JM, Zauls AJ, Wahlquist AH, Shirai K, Garrett-Mayer E, Gillespie MB, Day TA, Sharma AK. Low-dose weekly platinum-based chemoradiation for advanced head and neck cancer. Laryngoscope. 2010 Feb;120(2):236-42. doi: 10.1002/lary.20536. PMID: 19950378.

Al-Mamgani A, Verheij M, van den Brekel MWM. Elective unilateral nodal irradiation in head and neck squamous cell carcinoma: A paradigm shift. Eur J Cancer. 2017 Sep;82:1-5. doi: 10.1016/j.ejca.2017.05.035. Epub 2017 Jun 19. PMID: 28633075.

Bhattasali O, Han J, Thompson LDR, Buchschacher GL Jr, Abdalla IA, Iganej S. Induction chemotherapy followed by concurrent chemoradiation versus concurrent chemoradiation alone in the definitive management of p16-positive oropharyngeal squamous cell carcinoma with low-neck or N3 disease. Oral Oncol. 2018 Mar;78:151-155. doi: 10.1016/j.oraloncology.2018.01.031. Epub 2018 Feb 20. PMID: 29496043.

Chang PH, Yeh KY, Wang CH, Chen EY, Yang SW, Chou WC, Hsieh JC. Impact of metformin on patients with advanced head and neck cancer undergoing concurrent chemoradiotherapy. Head Neck. 2017 Aug;39(8):1573-1577. doi: 10.1002/hed.24793. Epub 2017 Apr 27. PMID: 28449193.

Elbers JBW, Al-Mamgani A, Paping D, van den Brekel MWM, Jóźwiak K, de Boer JP, Karakullukcu B, Verheij M, Zuur CL. Definitive (chemo)radiotherapy is a curative alternative for standard of care in advanced stage squamous cell carcinoma of the oral cavity. Oral Oncol. 2017 Dec;75:163-168. doi: 10.1016/j.oraloncology.2017.11.006. PMID: 29224815.

Iganej S, Beard BW, Chen J, Buchschacher GL Jr, Abdalla IA, Thompson LDR, Bhattasali O. Triweekly carboplatin as a potential de-intensification agent in concurrent chemoradiation for early-stage HPV-associated oropharyngeal cancer. Oral Oncol. 2019 Oct;97:18-22. doi: 10.1016/j.oraloncology.2019.07.016. Epub 2019 Aug 2. PMID: 31421466.

Mackiewicz J, Rybarczyk-Kasiuchnicz A, Łasińska I, Mazur-Roszak M, Świniuch D, Michalak M, Kaźmierska J, Studniarek A, Krokowicz Ł, Bajon T. The comparison of acute toxicity in 2 treatment courses: Three-weekly and weekly cisplatin treatment administered with radiotherapy in patients with head and neck squamous cell carcinoma. Medicine (Baltimore). 2017 Dec;96(51):e9151. doi: 10.1097/MD.0000000000009151. PMID: 29390445; PMCID: PMC5758147.

Müller von der Grün J, Martin D, Stöver T, Ghanaati S, Rödel C, Balermpas P. Chemoradiotherapy as Definitive Treatment for Elderly Patients with Head and Neck Cancer. Biomed Res Int. 2018 Jan 17;2018:3508795. doi: 10.1155/2018/3508795. PMID: 29581971; PMCID: PMC5822813.

Sommat K, Yit NL, Kwok LL. Comparison between 4-MV and 6-MV radiotherapy in T1N0 glottic cancer. Laryngoscope. 2017 May;127(5):1061-1067. doi: 10.1002/lary.26067. Epub 2016 May 30. PMID: 27237064.
